# The spectrum of psychological disorders in family members of patients suffering from delirium associated with critical illness: a prospective, observational study

**DOI:** 10.1038/s41598-024-53968-3

**Published:** 2024-02-24

**Authors:** Katarzyna Kotfis, Patrycja Maj, Aleksandra Szylińska, Maria Pankowiak, Elżbieta Reszka, E. Wesley Ely, Annachiara Marra

**Affiliations:** 1 Department of Anesthesiology, Intensive Care and Pain Management, Pomeranian Medical University, Szczecin, Poland; 2 Individual Psychotherapy Center, Warsaw, Poland; 3 Department of Cardiac Surgery, Pomeranian Medical University, Szczecin, Poland; 4 Student Science Club at the Department of Anesthesiology, Intensive Care and Pain Management, Pomeranian Medical University, Szczecin, Poland; 5 Critical Illness, Brain Dysfunction, and Survivorship (CIBS) Center, Center for Health Services Research, Nashville, TN USA; 6 Division of Allergy, Pulmonary and Critical Care, Department of Medicine, Vanderbilt University Medical Center, Nashville, TN USA; 7 Geriatric Research, Education and Clinical Center (GRECC) Service, Nashville Veterans Affairs Medical Center, Tennessee Valley Healthcare System, Nashville, TN USA; 8 Department of Neurosciences, Reproductive and Odontostomatological Sciences, University of Naples “Federico II”, Via Sergio Pansini, 5, 80131 Napoli, NA Italy

**Keywords:** Delirium, PICS, PICS-F, Family, Psychological disorders, Intensive care unit, Depression, Anxiety, Outcomes research, Health care

## Abstract

During intensive care unit admission, relatives of critically ill patients can experience emotional distress. The authors hypothesized that families of patients who are diagnosed with intensive care unit (ICU) delirium experience more profound depression and anxiety disorders related to stress than do families of patients without delirium. We performed a prospective observational single-center study including families of adult patients (age above 18 years) hospitalized in a 17-bed ICU of a university hospital for at least 48 h who completed research questionnaires at day 2 after admission and day 30 after initial evaluation using dedicated questionnaires (HADS, CECS, IES, PTSD-C). A total of 98 family members of patients hospitalized in the ICU were included in the final analysis (50 family members whose relatives were CAM-ICU positive (DEL+), and 48 family members of patients without delirium (DEL−)). No statistically significant differences in demographics and psychosocial data were found between the groups. In the follow-up 30 days after the first conversation with a family member, the mean PTSD score for the relatives of patients with delirium was 11.02 (Me = 13.0; SD = 5.74), and the mean score for nondelirious patients' family members was 6.42 (Me = 5.5; SD = 5.50; *p* < 0.001). A statistically significant increase in IES scores for family members of patients with delirium was observed for total PTSD (*p* = 0.001), IES—intrusion (*p* < 0.001), and IES—hyperarousal (*p* = 0.002). The prevalence of anxiety symptoms, depression, and posttraumatic stress disorder (PTSD) was higher in families of patients diagnosed with ICU delirium within 48 h of admission to the ICU. No factors increasing the depth of these disorders in family members of patients with ICU delirium were identified. Taking appropriate actions and thus providing families with appropriate support will contribute to the understanding of unfavorable emotional states, including anxiety, stress, depression, anger, agitation, or avoidance.

## Introduction

An unexpected patient admission to an intensive care unit (ICU) is stressful both for the patient and their family^1^. Family members of critically ill patients experience emotional distress with a spectrum of negative emotions, including anxiety, fear, guilt, frustration, anger, or irritation^2^, and are at high risk of anxiety and depressive conditions, including acute stress disorder, posttraumatic stress disorder (PTSD), depression, and complicated grief. Delirium is a disturbance of the state of consciousness with an acute onset and a variable course, accompanied by a change in perception that often coexists with the underlying disease, but importantly, it increases mortality, prolongs hospitalization, and may create a predisposition to cognitive impairment after ICU discharge^3^. ICU delirium is a commonly neglected and underdiagnosed manifestation of organ dysfunction in the ICU with a severe influence on patients’ dignity, their family, and relatives^4^. Untreated and worsening delirium is associated with increased mortality and the development of long-term physical disability and cognitive and psychiatric disorders, including symptoms of posttraumatic stress disorder (PTSD), which are now recognized as postintensive care syndrome (PICS). People can experience a range of different reactions following a traumatic experience, which subside over time. Those who still experience symptoms may be diagnosed with PTSD. PTSD symptoms usually appear within 3 months of the traumatic event but may also have a delayed onset. To meet the criteria for PTSD, symptoms must persist for longer than 1 month and negatively impact aspects of daily life, such as relationships or work. The symptoms also cannot be related to taking medications, using psychoactive substances or any other disease. Individuals suffering from PTSD experience re-experiencing symptoms, avoidance, arousal and reactivity symptoms, cognitive and mood symptoms^4^. PICS is a health problem that remains after a serious illness, which occurs during the patient's stay in the ICU and may continue after leaving the hospital. PICS may manifest as ICU-acquired weakness, cognitive dysfunction, and other mental health problems, e.g. problems with falling or staying asleep, nightmares and unwanted memories, feeling depressed and anxious^5^. These symptoms can affect both patients (PICS) and families (PICS-F)^5^. Studies have shown that 33–49% of family members experience PTSD^6,7^. A high proportion of family members present with symptoms of anxiety (70%) and depression (35%)^8,9^, disturbances that lead to disruption of previous family lifestyle and problems in the workplace. The burden on families should be assessed routinely and requires preventive strategies and specific treatments^10^.

Research has shown that the implementation of nonpharmacological interventions to prevent delirium, including interventions provided by relatives, such as reading newspapers, news, family photographs or bringing items for optimal reorientation^5^, can reduce the incidence of delirium. Therefore, the family^6^ has an important role in the care of ICU patients^5^. Family caregivers provide important emotional support during and after a critical illness and help the ICU team in making decisions and acting for the patient. Health-care workers should play a supportive role through effective communication with families and relatives, indicating action directions, ways of coping with stress, building attitudes and actions aimed at regulating emotions and spiritual needs^11^. Those actions have a significant impact on reducing the occurrence of PTSD or depression in the patient's family members^12^.

Hospitalization of critically ill patients requiring intensive care is challenging for family members who could experience stress, anxiety, depression, sleep disturbances and symptoms of posttraumatic stress disorder^13^. Just as the presence of the family is important for patients, both personal and virtual or remote contact is equally important for the family members themselves, as it enables them to cope with a difficult situation^14^.

Psychological disorders resulting from ICU delirium in family members of patients treated in the intensive care units are frequent and at the same time little-known in Poland and other Eastern European countries. Therefore, efforts should be made to identify and adequately address this topic. Patients suffering from delirium experience sudden cognitive and behavioral changes that can be traumatic and stressful not only for the patient but also for caregivers who experience severe distress related to delirium^15^. In order to quantify the size for the problem we performed a qualitative, questionnaire-based study involving first line relatives of patients diagnosed with ICU delirium. This study aimed to 1) compare the incidence of symptoms of anxiety, depression and posttraumatic stress disorder (*PTSD*) in family members of patients with and without ICU delirium during hospitalization in the intensive care unit within 48 h of admission and 30 days after the initial evaluation (regardless of the fact that patient has been or has not been discharged from the hospital), 2) analyze the range of factors (i.e., demographic, social, emotional) affecting the depth of these disorders in family members, and 3) determine the emotional needs of families of patients treated in the ICU.

To explore the above research aims, the authors hypothesized that families of patients with ICU delirium experience more profound depression and anxiety disorders than families of patients without delirium.

## Methods

A prospective observational single-center study was performed in compliance with The Declaration of Helsinki, ICH Guideline for Good Clinical Practice E6(R2) after the approval of the Institutional Ethics Committee of the Pomeranian Medical University (Approval No. KB-0012/85/19, dated z 1.04.2019).

The study included families of adult patients (age 18 years and above) hospitalized in a 17-bed ICU of a university hospital for at least 48 h. The family members who agreed to participate in the study were informed about the course and purpose of the study, had time to ask questions and agreed to sign written consent to participate in the study and complete research questionnaires initially and 30 days afterwards. The patients’ depth of sedation was assessed by ICU physicians with the Richmond Agitation Sedation Scale (RASS), and screening for delirium was performed every 8 h with the use of the Confusion Assessment Method for Intensive Care Unit (CAM-ICU). The study was conducted between July 2019 and December 2022 at the Pomeranian Medical University in Szczecin using validated questionnaires, including Hospital Anxiety and Depression Scale (HADS), the Emotional Control Scale (CECS), The clinical version of Post-traumatic Stress Disorder (PTSD-C), and the Impact Event Scale (IES). As the study was performed during the COVID-19 pandemic, families of patients with an underlying diagnosis of SARS-CoV-2 infection were invited to participate in the study.

### Ethical approval

This study was approved by the Bioethics Committee at the Pomeranian Medical University in Szczecin (approval no. KB-0012/85/19, dated 1.04.2019).

### Consent to participate

All participants gave their informed consent.

## Study measurements

A specially designed diagnostic survey was conducted using a questionnaire technique. It included the Hospital Anxiety and Depression Scale (HADS), the Emotional Control Scale (CECS), The clinical version of Post-traumatic Stress Disorder (PTSD-C), and the Impact Event Scale (IES), performed within 48 h of ICU delirium diagnosis after the patient's admission into the ICU and then 30 days after the initial interview during a meeting with the family or through a telephone interview. The data was collected on a printed paper document as approved by the Institutional Ethics Committee (IEC).

A specially constructed questionnaire was prepared to analyze emotional disorders and possible risk factors for emotional disorders in families, such as demographic factors (age, sex, degree of kinship), psychosocial factors (education, financial status), factors related to the patient's underlying disease, the method of communication with the medical staff (knowing the prognosis, explaining treatment methods, providing information about the progress of treatment, receiving reliable answers) or information related to educating the family about ICU delirium. Many of these factors have been shown by Jezierska et al. as risk factors for the development of PICS-F^16^.

HADS is a screening tool designed to recognize the presence of depressed mood and anxiety with an assessment of their intensity. The Cronbach’s alpha for HADS subscale for anxiety ranged from 0.68 to 0.93 (mean 0.83) and for depression from 0.67 to 0.90 (mean 0.82)^17^. The modification consists of 16 questions that respondents must complete with answers presented on a four-degree scale for each question. Eight of these statements are used to measure anxiety, and another 8 are used to measure depression. Each item is then rated on a scale of 0 to 3 points. The cutoff threshold is 7 points (for depression) and 7 points (for anxiety). CECS contains 21 statements concerning the three basic emotions: anger, depression, and anxiety^18^. It is used to subjectively evaluate the respondent's control of these emotions in difficult situations. The examined person determines the frequency of the given way of expressing emotions on a 4-degree scale, from 1 (almost never) to 4 (almost always). Scores are calculated separately for each subscale. By summing all the results, the overall emotion control index is determined, which ranges from 22 to 84 points. A higher score is associated with a greater tendency to suppress negative emotions.

The clinical version of Post-traumatic Stress Disorder contains criteria for the diagnosis of PTSD and enables the recognition of various forms of the disorder. The Cronbach’s alpha for this scale is 0.94^19^. The basic criterion is a person's confirmed exposure to a traumatic event that threatens them or others with death or injury and leads to intense fear, terror, and helplessness. The questionnaire contains 22 questions to which the participants answer affirmatively or negatively (YES/NO).

The Impact of Event Scale-Revised (IES-R) aims to determine the current, subjective sense of discomfort associated with stressful events^20^. The scale describes three aspects of PTSD: 1. Intrusion–recurring images, dreams, thoughts, or perceptual impressions related to the trauma; 2. Hyperarousal–increased vigilance, fear, impatience, difficulty concentrating; and 3. Avoidance–a strong need to eliminate thoughts, emotions or conversations related to the trauma. The scale demonstrates a high internal consistency, with a Cronbach’s alpha of 0.78 for intrusion and 0.42 for avoidance^20^. The IES-R scale contains 22 statements describing the symptoms of stress experienced in the last 7 days in relation to the experienced traumatic event. The evaluation is made on a 5-degree Likert scale (0, 1, 2, 3, 4). The Negative Mood Scale was used to assess the level of negative emotions and consists of 7 adjectives expressing a negative mood, i.e., 1. Nervousness, 2. Fear, 3. Anxiety, 4. Anger, 5. Uncertainty, 6. Helplessness, and 7. Depression. The intensity of these emotions is described on a 5-degree scale: none–1, slight–2, moderate–3, high–4, very high–5.

ICU delirium was diagnosed by the ICU physician using the RASS and CAM-ICU scales. The CAM-ICU was validated in 2001 by Ely et al., showing sensitivity of 93% and 100% and specificity of 98% and 100%^21^. The patient's level of consciousness is assessed using the RASS scale. The assessment is made on a point scale from − 5 to + 4, which determines the patient's state of sedation and arousal. Negative scale values indicate deep sedation, and increasing positive values indicate patient agitation^22^. The CAM-ICU scale is not used when the patient's RASS score is between − 4 and − 5 (i.e., in a state of deep sedation and coma)^23^. The CAM-ICU is used when RASS is equal to or greater than − 3 (from − 3 to + 4) and it is possible to assess the changes in the patient's mental status. Then, by asking simple, logical questions, the patient's inattention, altered level of consciousness and disorganized thinking are evaluated.

### Statistical methods

Statistical analysis was carried out with Statistica 13 software (StatSoft, Inc. Tulsa, OK, USA). Continuous variables are summarized as the means, medians, and standard deviations, and qualitative variables are summarized as numbers and percentages. The Shapiro‒Wilk test was used to assess the normality of variable distribution. Homogeneity of variance was verified by Levene's test. Using the Mann‒Whitney U test, continuous variables were compared between a group of family members of patients with delirium (DEL+) and without delirium (DEL−); if the distribution of these variables was abnormal and in the case of a normal distribution, Student's t test was used. Single- and multifactor logistic regression analyses were performed. Logistic regression results were presented as odds ratios and confidence intervals for the odds ratio. Multivariate analysis was adjusted for demographic data (gender and age). A comparison of the incidence of symptoms of anxiety, depression, and posttraumatic stress disorder (PTSD) in family members of patients with and without ICU delirium was performed with the Mann‒Whitney U test or Student's t test. The analysis of the range of factors affecting the depth of disorders in family members was performed with multivariate logistic regression analysis. The emotional needs of families of patients treated in the ICU was performed with used chi-square test. A *p* value *p* < 0.05 indicates statistically significant differences.

## Results

### An analysis of demographics and psychosocial data

**Figure 1:**
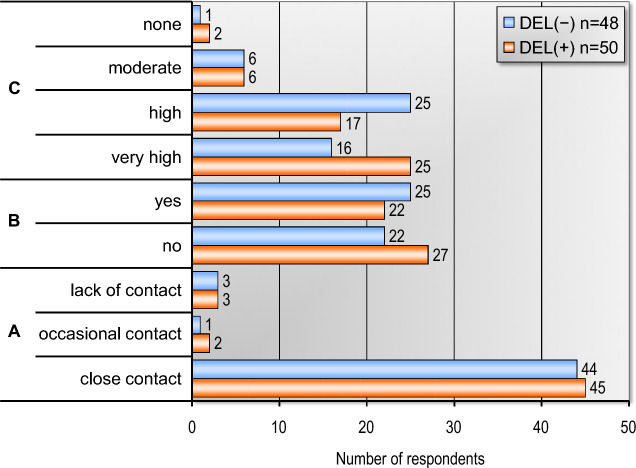
Psychosocial data of the respondents. (**A**)—Previous relationship with a patient. (**B**)—Living with the patient in one household. (**C**)—The degree to which the current illness of a loved one affects relationships with other people.

A total of 98 family members of patients hospitalized in the ICU were included in the final analysis, with 50 family members of the CAM-ICU-positive (DEL+) group and 48 family members of the CAM-ICU-negative (DEL−) group who gave informed consent to complete the research questionnaires. All participants were 18 years old or above and of both sexes. No statistically significant differences in demographics and psychosocial data were found between family members of the two groups (Table 1, Fig. 1).

**Table 1:** Demographic data of the respondents.

Variables	DEL (+) n = 50	DEL (−) n = 48	*p*
Age [years], mean ± SD; Me	54.00 ± 14.60; 60.0	55.17 ± 13.07; 56.0	0.712*
Gender, n (%)			
Female	37 (74%)	34 (70.8%)	0.726
Male	13 (26%)	14 (29.2%)	
Education, n (%)			
Primary	6 (12%)	4 (8.3%)	0.967
Vocational	8 (16%)	9 (18.75%)	
Secondary	15 (30%)	13 (27.1%)	
University	20 (40%)	21 (43.75%)	
Postgraduate	1 (2%)	1 (2.1%)	
Marital status, n (%)			
Single	4 (8%)	2 (4.4%)	0.769
Married/partner	38 (76%)	36 (80%)	
Separated/divorced	8 (16%)	7 (15.6%)	
Degree of kinship, n (%)			
Mother/father	4 (8%)	2 (4.2%)	0.576
Sister/brother	2 (4%)	3 (6.25%)	
Daughter/son	21 (42%)	25 (52.1%)	
Spouse/partner	22 (44%)	17 (35.4%)	
Grandchild	1 (2%)	0 (0%)	
Aunt/uncle	0 (0%)	1 (2.1%)	

SD, Standard deviation; Me, Median; n, Number of patients; *p*, Statistical significance. *U Mann‒Whitney test.

### An analysis of data obtained within 48 h of ICU admission

The analysis showed that families of patients with ICU delirium were characterized by significantly higher levels of anxiety, depression control, PTSD, introversion. A significant increase in HADS (assessing in-hospital anxiety and depression) and CECS scores (assessing emotion control) was observed in the family members of patients diagnosed with ICU delirium. Statistically significant differences were obtained in the HADS scale in questions on anxiety (*p* = 0.014), with the family members who were diagnosed with delirium having a mean score of 9.96 ± 3.16, while the family members of patients without delirium had a mean score of 8.17 ± 3.92. In terms of emotional control (CECS scale), statistically significant differences were obtained in questions regarding depression (*p* = 0.019). The family members of ICU patients diagnosed with delirium scored a mean score of 17.70 ± 4.48 points, while the family members of patients without delirium scored 15.69 ± 3.87 points. The data are reported in Table 2.

**Table 2:** Evaluation of hospital anxiety, depression, and emotional control of respondents.

Variable	DEL (+) n = 50			DEL (−) n = 48			*p*
	${\overline{\text{x}}}$	Me	± SD	${\overline{\text{x}}}$	Me	± SD	
HADS–fear	9.96	10.0	3.16	8.17	7.5	3.92	0.014*
HADS–depression	10.56	10.0	3.12	10.15	9.0	4.32	0.277
CECS–emotion control–Anger	17.74	18.0	4.38	16.60	17.0	4.77	0.222*
CECS–emotion control–depression	17.70	18.0	4.48	15.69	16.0	3.87	0.019*
CECS–emotion control–anxiety	16.82	17.5	4.16	16.04	16.0	4.50	0.464

SD, Standard deviation; ${\overline{\text{x}}}$, Mean; Me, Median; n, Number of patients; *p*, Statistical significance. Analysis: Mann‒Whitney U test. * Student’s t test was used.

Multivariate logistic regression analysis (Table 3) for the HADS and CECS scales confirmed the relationship between the occurrence of ICU delirium in patients treated in the intensive care unit and the anxiety experienced by their relatives on the HADS scale (OR = 1.137, *p* = 0.042) and depression on the CECS scale (OR = 1.121, *p* = 0.035). There was no association between the two groups for HADS depression (*p* = 0.583), CECS anger (*p* = 0.222) and CECS anxiety (*p* = 0.373).

**Table 3:** Multivariate logistic regression analysis for HADS and CECS at 48 h.

Evaluation at 48 h	*p*	OR	Confidence OR − 95%	Confidence OR + 95%
HADS–fear	0.042	1.137	1.005	1.287
HADS–depression	0.693	1.023	0.915	1.143
CECS–emotion control–anger	0.249	1.055	0.963	1.157
CECS–emotion control–depression	0.035	1.121	1.008	1.246
CECS–emotion control–fear	0.486	1.035	0.940	1.139

OR, Odds ratio; *p*, Statistical significance.

Before their relatives completed the second part of the questionnaire, 10 of the delirious and 9 of the nondelirious patients died (*p* = 0.876).

### An analysis of data obtained 30 days after initial evaluation

In the analysis of the follow-up at 30 days after the first conversation with a family member, a significant increase in PTSD and IES scores was observed in loved ones of patients diagnosed with delirium (Table 4). The mean score for these relatives was 11.02 (Me = 13.0; SD = 5.74), and the mean score for nondelirious patients' family members was 6.42 (Me = 5.5; SD = 5.50). The result was statistically significant (*p* < 0.001). In the assessment of the IES scale, a statistically significant increase in IES scores was also observed–total PTSD (*p* = 0.001), IES—intrusion (*p* < 0.001), IE—hyperarousal (*p* = 0.002).

**Table 4:** Evaluation of posttraumatic stress disorder and the impact of events at 30 days after initial evaluation in respondents.

Evaluation at 30 days	DEL (+) n = 40			DEL (−) n = 39			*p*
	${\overline{\text{x}}}$	Me	± SD	${\overline{\text{x}}}$	Me	± SD	
IES–PTSD total	2.36	2.6	0.76	1.87	1.9	0.68	0.001
IES–intrusion	2.53	2.8	0.81	1.84	1.9	0.69	< 0.001*
IES–hyperarosual	2.44	2.8	0.96	1.90	2.0	0.80	0.002
IES–avoidance	2.09	2.1	0.75	1.87	1.9	0.76	0.158*
PTSD	11.02	13.0	5.74	6.42	5.5	5.50	< 0.001

SD, Standard deviation; ${\overline{\text{x}}}$, Mean; Me, Median; n, Number of patients; *p*, Statistical significance. Analysis: Mann‒Whitney U test. * Student’s t test was used.

Multivariate logistic regression analysis for the IES scale and the PTSD scale confirmed the association with the occurrence of delirium in ICU patients and IES–PTSD total (OR = 2.485, *p* = 0.004), IES—intrusion (OR = 3.391, *p* < 0.001), IES—hyperarousal (OR = 1.969, *p* = 0.007) and PTSD (OR = 1.169, *p* < 0.001), as shown in Table 5.

**Table 5:** Multivariate logistic regression analysis for PTSD and IES at 30 days.

Variable	*p*	OR	Confidence OR − 95%	Confidence OR + 95%
IES PTSD total	0.004	2.485	1.344	4.592
IES–intrusion	< 0.001	3.391	1.779	6.462
IES–hyperarosual	0.007	1.969	1.201	3.228
IES–avoidance	0.221	1.419	0.810	2.488
PTSD	< 0.001	1.169	1.077	1.270

OR, Odds ratio; *p*, statistical significance.

### An assessment of the feelings of the patients' family members

Table 6 shows data regarding the feelings of family members. There were no statistically significant differences between the two groups in experiencing somatic symptoms (headaches, abdominal pain, palpitations) while experiencing emotions (*p* = 0.146), but they were common in both groups (72% [DEL+] and 59% [DEL−]). Similarly, there were no statistically significant differences between the groups in the assessment of the support received. To the question "Did you receive support from relatives (family, friends) in connection with the serious illness of a loved one?" Most respondents in both groups answered affirmatively: 84% (DEL+) and 72.5% (DEL−) (*p* = 0.569). The same applies to the question "Did you receive support from ICU medical personnel in connection with a serious illness of a loved one?". Most respondents in both groups answered affirmatively: 82% (DEL+) and 64% (DEL−), with no statistically significant differences (*p* = 0.184).

**Table 6:** Assessment of the feelings of the patients' family members.

Variables		DEL (+) n = 50	DEL (−) n = 48	*p*
How intensive were somatic symptoms (headaches, abdominal pain, palpitations) that you experienced in relation to perceived emotions?	Very severe	17 (34%)	9 (18.8%)	0.146
	Severe	19 (38%)	19 (39.6%)	
	Moderate	10 (20%)	18 (37.5%)	
	None	4 (8%)	2 (4.2%)	
Did you receive support from relatives (family, friends) in connection with the serious illness of a loved one?	No support	0 (0%)	1 (2.1%)	0.569
	Little support	1 (2%)	3 (6.25%)	
	Moderate support	7 (14%)	9 (18.75%)	
	High support	19 (38%)	17 (35.4%)	
	Very high support	23 (46%)	18 (37.5%)	
Did you receive support from ICU medical personnel in connection with a serious illness of a loved one?	No support	0 (0%)	0 (0%)	0.184
	Little support	2 (4%)	5 (10.42%)	
	Moderate support	7 (14%)	12 (25%)	
	High support	27 (54%)	17 (35.42%)	
	Very high support	14 (28%)	14 (29.17%)	
Did you obtain information from the medical staff about the patient's treatment?"	No response	0 (0%)	0 (0%)	0.025
	Little response	0 (0%)	0 (0%)	
	Moderate response	5 (10%)	12 (25%)	
	High response	27 (54%)	14 (29.2%)	
	Very high response	18 (36%)	22 (45.8%)	
Did you understand the information provided about the patient's treatment?	No	0 (0%)	0 (0%)	0.601
	In small extent	2 (4%)	1 (2.1%)	
	Moderately	12 (24%)	17 (35.4%)	
	Mostly	21 (42%)	16 (33.3%)	
	Definitely yes	15 (30%)	14 (29.2%)	
Did the medical staff answer your questions?	No	0 (0%)	0 (0%)	0.155
	In small extent	1 (2%)	1 (2.1%)	
	Moderately	5 (10%)	4 (14.6%)	
	Mostly	27 (54%)	15 (31.25%)	
	Definitely yes	17 (34%)	25 (52.1%)	
To what extent did your emotional reaction to the patient's delirium change anything in your life?	No	2 (4%)	5 (10.6%)	0.030
	In small extent	4 (8%)	8 (17%)	
	Moderately	9 (18%)	15 (31.9%)	
	Mostly	23 (46%)	16 (34%)	
	Definitely yes	12 (24%)	3 (6.4%)	
Did you have a need to do something (or not to do anything) under the influence of the emotion?	No	3 (6.25%)	6 (12.5%)	< 0.001
	In small extent	2 (4.17%)	5 (10.4%)	
	Moderately	4 (8.33%)	20 (41.7%)	
	Mostly	33 (68.75%)	10 (20.8%)	
	Definitely yes	6 (12.5%)	7 (14.6%)	

ICU, Intensive care unit; SD, Standard deviation; Me, Median; n, Number of patients; *p*, Statistical significance. Analysis used: chi-square test.

Families of patients admitted to the ICU had no problem obtaining information from the medical staff regarding the patient's condition. Regarding the question "Did you obtain information from the medical staff about the patient's treatment?" Most of the participants, i.e., 90% in the (DEL+) group, answered ‘mostly’ (54%, n = 27) or ‘definitely yes’ (36%, n = 18). Similarly, in the (DEL−) group, 75% of the respondents answered affirmatively: ‘largely' 29% (n = 14) or 'definitely yes' 46% (n = 22), *p* = 0.025. To the question "Do you understand the information provided about the patient's treatment?" Most respondents in both groups answered affirmatively: 72% DEL+ and 64% DEL− (*p* = 0.601).

The availability of the medical staff toward the ICU patient's family is an important aspect. In response to the question "Does medical staff answer your questions?" There was no significant difference between the two subgroups (*p* = 0.155). An affirmative answer was declared by 88% of respondents from the (DEL+) group and 83% from the (DEL−) group.

A statistically significant difference between the groups was shown regarding the impact of the patient's delirium on the emotional reaction of a family member (*p* = 0.030). To the question "To what extent did your emotional reaction to the patient's delirium change anything in your life?" in the (DEL+) group, 70% of the respondents chose an affirmative answer: ‘mostly’-46% (n = 23), ‘definitely yes’ 24% (n = 12). In the (DEL−) group, 34% (n = 16) of the respondents answered ‘mostly' and 32% (n = 15) chose the answer 'moderately'.

A statistically significant difference occurred between the members of the (DEL+) and (DEL−) families in response to the question "the need to do something (or not to do anything) under the influence of the emotion"–the answer "mostly" was chosen by as many as 33 (69%) family members of patients with ICU delirium and only 10 subjects (21%) in the group of family members of nondelirious patients (*p* < 0.001).

**Figure 2:**
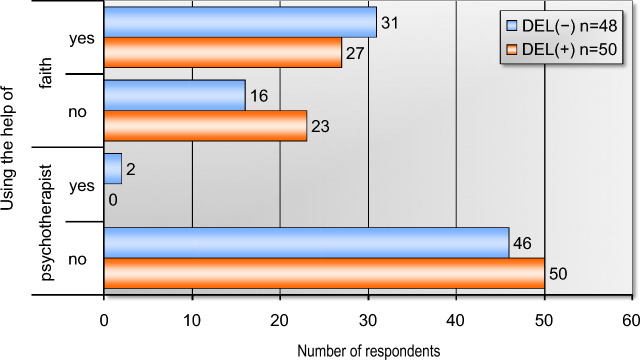
Using the support of psychotherapist and faith by respondents.

Data presented in Fig. 2 shows that a comparable number of respondents from both groups used the support of their faith—31 in the (DEL+) group and 27 in the (DEL−) group. Only 2 respondents from the whole group (DEL+) used the support of a psychotherapist to deal with the current emotional situation (Fig. 2).

## Discussion

The aim of this study was to compare the incidence of symptoms of anxiety, depression, and posttraumatic stress disorder (PTSD) in family members of patients with and without delirium during treatment in the intensive care unit and within 30 days after discharge. We found that family members of patients with delirium showed significantly higher levels of anxiety, depression control, PTSD, intrusion, and agitation compared to family members of patients without delirium. Based on the research tool used (e.g., HADS and CECS questionnaires), an increase in indications of negative emotional effects in family members of patients diagnosed with severe delirium can be confirmed. On the HADS scale, family members with delirium had higher mean scores on questions regarding anxiety than family members of nondelirious patients (Tables 2, 3). Anxiety, nervousness, depression, or PTSD were more common in family members whose relatives suffered from acute central nervous system disorders during intensive care unit admission (Tables 4, 5).

The impact of treating a loved one in the ICU on the increased risk of PTSD in the family of such a person has been confirmed by numerous studies^1,7^. Unfortunately, PTSD symptoms may persist even for a longer period (up to several years) after ICU discharge, especially when the outcome is unfavorable and ends in the patient's death^24^.

In addition to the knowledge resulting from our research, comparative information is provided by other authors. It is extremely important to emphasize that the care of a critical illness survivor after ICU discharge is challenging to the family that needs to provide specialized treatment to provide a loved one with appropriate care and support in everyday activities and targeted physiotherapy. At the same time, the situations a person faces indicate the depth of trauma and depression and the need to provide specialist support. As shown by Płaszewska-Żywko, such support, required from the medical staff (doctors, nurses, physiotherapists), is often recommended to reduce the risk of mental problems in the patient's family related to anger, anxiety, depression, or PTSD^25^. Jezierska et al. indicate that a particularly strong impact on the family’s emotional state has been demonstrated when ICU patients are children and people who require support from their mothers^16^.

These results concerning the mental burden of families are not limited to studies confirming the influence of delirium but are relevant to family members of a patient hospitalized in the ICU regardless of the delirium status^26,27^. Similarly, the study conducted by Heesakkers et al. also showed a huge burden on relatives of ICU patients during and after the COVID-19 pandemic^28^. In this study, the IES score and the HADS score were significantly higher among family members who were present at the event leading to admission to the ICU, and therefore, this knowledge should be considered when caring for family members^29^. What is more and underlined by Rosgen et al. the support necessary for families of people with delirium can be of different nature^30^. Lange and White differed in their approach, but both authors emphasized the importance of shared family decision making in the process of patient care^31,32^. The highest support is allowing the family to perform some patient-related care activities. Such actions are commonly used in Western countries^33^, relatively less frequently in Poland and other Eastern European countries. At the same time, Carbone recommends educating the families because, as it turns out, the increased awareness of the situation’s development and emotional burden associated with a crisis leads to alleviating the symptoms of such conditions^34^.

In terms of emotion control (CECS scale) in questions about depression, the results obtained by the authors indicated statistically significant differences, confirming the view that the feeling of depression is closely related to the diagnosis of delirium in a family member treated in ICU conditions. As is in line with the observation of other authors, including Poulin et al. who also indicated a relatively high probability of depression in family members of patients with delirium^35^.

The stress remains high after the patient with delirium is discharged from the ICU, persistently influencing the family members as is visibly in this study and highlighted by other authors. This requires solutions, i.e., Bohart et al. stated that ICU staff must equip patients and their family members with some necessary skills to support specific family members in fulfilling the role of patient advocates and supporters^36^. Relatives and friends should be involved in managing patients with ICU delirium by supporting them emotionally, reorientating, giving insight into their needs, and providing patients with personal sensory devices (hearing aids or glasses) or familiar items, scents, or music^1,37–39^. This can only be done with the active involvement of all staff through communication and information sharing, including the involvement of social media to help people cope with stress. Clinically robust research is needed to identify effective social media strategies for caregivers of critically ill patients^40^.

### Limitations

Our findings should be interpreted in the context of several limitations. This study is not without limitations. First, this is a single-center study; therefore, local factors may preclude its generalizability. Research based on questionnaires is limited to previously constructed, available scales. Operationalization of the adopted criteria led to the conclusion that it is necessary to perform the diagnosis at two points in time, i.e., directly after admission to the ICU and 30 days after. It seems that the time distance between these two time points is too large to identify all the psychological problems of families, and perhaps in future analyses, additional assessment scales should be included, or the depth of disorders should be examined more frequently (after 7, 14 or 21 days) from the initial hospitalization of the patient in the ICU. Therefore, using the selected tools, the examined person would be asked to assess the reaction to the traumatic event in additional moments, which would deepen the comparative diagnosis. Moreover, there may be residual confounding factors in the regression models used in this study that have not been previously identified and controlled for.

## Conclusions

When comparing family members of critically ill patients with and without ICU delirium, the prevalence of anxiety symptoms, depression, and posttraumatic stress disorder (PTSD) within 30 days after the onset of the initial illness is higher in families of patients diagnosed with ICU delirium during their ICU stay. No factors increasing the depth of these disorders in family members of patients with ICU delirium were identified. Assessment of the emotional needs of the family members of critically ill patients treated in the ICU leads to the conclusion that members of critically ill adults who were diagnosed with ICU delirium within 48 h of ICU admission frequently experience clinically significant anxiety.

The presented results and conclusions may be of practical use. Prevention of the negative consequences of staying in the ICU in the families of patients with ICU delirium assumes equipping the ward staff with knowledge about the mental burden of the patients' families, their needs, and the possibilities of meeting them both during the patient's stay in the ward and at home. Taking appropriate actions and thus providing families with appropriate support will contribute to understanding unfavorable emotional states, such as anxiety, stress, depression, anger, agitation, or avoidance.

## Data Availability

The data that support the findings of this study are available on request from the first author KK.

## Abbreviations

CAM-ICU: Confusion assessment method for the intensive care unit
CECS: Courtauld emotional control scale
DEL(−): Patients without ICU delirium
DEL(+): Patients with ICU delirium
HADS: Hospital anxiety and depression scale
ICU: Intensive care unit
ICU Delirium: Intensive care unit delirium
IES: Impact event scale
IES-R: Impact event scale-revised
Me: Median
n: Number of patients
OR: Odds ratio
*p*: Statistical significance
PICS: Postintensive care syndrome
PICS-F: Postintensive care syndrome–family
PTSD: Post-traumatic stress disorder
PTSD-C: Clinical version of post-traumatic stress disorder
RASS: Richmond agitation, sedation scale
SD: Standard deviation
${\overline{\text{x}}}$: Mean; intermediate value
